# Evaluation of the antibacterial activity of selected Kenyan medicinal plant extract combinations against clinically important bacteria

**DOI:** 10.1186/s12906-023-03939-4

**Published:** 2023-04-03

**Authors:** Elizabeth A Odongo, Peggoty C Mutai, Beatrice K Amugune, Nelly N Mungai, Mary O Akinyi, Julia Kimondo

**Affiliations:** 1 Department of Pharmaceutical Chemistry, Pharmaceutics & Pharmacognosy, University of Nairobi, P.O. Box 19676-00202, Nairobi, Kenya; 2 Department of Pharmacy, Kenya Methodist University, P.O. Box 19676-00202, Meru, Kenya; 3 Department of Pharmacognosy, Jomo Kenyatta University of Agriculture Technology, P.O. Box 62000- 00202, Nairobi, Kenya

**Keywords:** Medicinal plants, Antibacterial, Synergistic, Traditional medicine

## Abstract

**Background:**

Infectious diseases are a major global public health concern as antimicrobial resistance (AMR) currently accounts for more than 700,000 deaths per year worldwide. The emergence and spread of resistant bacterial pathogens remain a key challenge in antibacterial chemotherapy. This study aims to investigate the antibacterial activity of combined extracts of various Kenyan medicinal plants against selected microorganisms of medical significance.

**Methods:**

The antibacterial activity of various extract combinations of *Aloe secundiflora, Toddalia asiatica, Senna didymobotrya* and *Camelia sinensis* against *Staphylococcus aureus*, *Escherichia coli*, *Pseudomonas aeruginosa*, *Klebsiella pneumoniae* and *Methicillin Resistant Staphylococcus aureus* was assessed using the agar well diffusion and the minimum inhibitory concentration *in-vitro* assays. The checkerboard method was used to evaluate the interactions between the various extract combinations. ANOVA test followed by Tukey’s post hoc multiple comparison test was used to determine statistically significant differences in activity (P < 0.05).

**Results:**

At concentrations of 100 mg/ml (10,000 µg/well), the different combinations of the aqueous, methanol, dichloromethane and petroleum ether extracts of the selected Kenyan medicinal plants revealed diverse activity against all the test bacteria. The combination of methanolic *C. sinensis* and *A. secundiflora* was the most active against *E. coli* (14.17 ± 0.22 mm, diameter of zones of inhibition (DZI); MIC 2500 µg/well). The combination of methanolic *C. sinensis* and *S. didymobotrya* was the most active against *S. aureus* (16.43 ± 0.10 mm; MIC 1250 µg/well), *K. pneumonia* (14.93 ± 0.35 mm, DZI; MIC 1250 µg/well), *P. aeruginosa* (17.22 ± 0.41 mm, DZI; MIC 156.25 µg/well) and *MRSA* (19.91 ± 0.31 mm, DZI; MIC 1250 µg/well). The Minimum Inhibitory Concentration of the different plant extract combinations ranged from 10,000 µg/ well to 156.25 µg/well. The ANOVA test indicated statistically significant differences (P < 0.05) between single extracts and their combinations. The fractional inhibitory concentration indices (FICI) showed that the interactions were either synergistic (10.5%), additive (31.6%), indifferent (52.6%), or antagonistic (5.3%) for the selected combinations.

**Conclusion:**

This study findings validate the ethnopractice of selectively combining medicinal plants in the management of some bacterial infections in traditional medicine.

## Background

Antibiotics have made a considerable contribution to the control of infectious diseases that have over time contributed to human morbidity and mortality for most of human existence [1]. In spite of the existing range of conventional antimicrobial agents in clinical use, antimicrobial resistance (AMR) remains a constant threat with regular antibiotic use [2, 3]. Among bacterial infections, the so-called “ESKAPE” pathogens have caused the most concerns based on their prevalence and overall mortality [4]. Failure to take the appropriate measures to combat the progress of antimicrobial resistance may result in the loss of approximately 10 million lives and cost about US$100 trillion per year by 2050 [5]. The significant gaps in the surveillance of antimicrobial resistance coupled with a lack of quality data on the impact of antimicrobial resistance is a common observation in most African countries [5].

There is a continuous need to develop new medicines that are capable of overcoming microbial resistance. Approximately, 30 to 40% of the commercially available antimicrobial drugs are from natural products and primarily from microbial origins [6]. Plants are a promising alternative in the search for new antimicrobials based on their utilization in traditional medicine for the management of bacterial diseases and potential to provide an unlimited range of chemical compounds for exploration [6, 7]. Over 1340 plants possess defined antimicrobial activity and about 30,000 antimicrobial compounds have been isolated from plants [8].

Drug combination is a recognized approach in both traditional and conventional medicine systems. It is based on synergistic interactions to improve the therapeutic efficacy and lifespan of drugs [9–11]. For example, locals around the Lake Victoria basin in Tanzania reportedly utilize multi-plant extracts in the management of secondary opportunistic infections [12]. Polyherbalism is also famous in Ayurveda [13]. While the combination of conventional drugs is a common practice and a successful tactic in the management of drug resistant microorganisms, the outcome of combinations between herbal drugs remains obscure due to limited scientific appraisal [14].

Based on a previous systematic review on the antibacterial activity of Kenyan medicinal plants, *Camelia sinensis, Aloe secundiflora, Toddalia asiatica* and *Senna didymobotrya* were selected for pharmacological assay as they exhibited high mean inhibition zone values and/ or low minimum inhibitory concentration (MIC) values [15, 16].

*Camelia sinensis* L. (Theaceae) is a common evergreen shrub widely grown in many parts of the world. It is used as an astringent, stimulant, diuretic and de-flatulent in traditional medicine [17]. It has antioxidant, antimicrobial, cholesterol lowering and cardio protective effects [18]. The bioactive constituents include caffeine, L-theanine and polyphenols/flavonoids, proteins, minerals, vitamins, and amino acids [17, 19].

*Aloe secundiflora* Engl. (Asphodelaceae) widely famed for its medicinal and cosmetic properties is the most commonly used Aloe species in Kenya [20]. It remedies constipation, sore throat and promotes wound healing [15]. The chemical constituents comprise tannins, terpenoids and flavonoids [21].

*Toddalia asiatica* L. (Rutaceae) is a traditional remedy for coughs, dysentery and malaria [22]. It has anti-inflammatory, analgesic, hemostatic coagulation anti-tumor effects. The main chemical constituents are coumarins and alkaloids [23].

*Senna didymobotrya* (Fres.) Irwin & Barneby (Fabaceae) is abundant across East Africa and the traditional preparations relieve diarrhea, malaria and ringworm [24]. Its pharmacological effects include antibacterial, antifungal and antioxidant [25]. The chemical constituents consist of steroids, terpenoids, anthraquinones, tannins, saponins, glycosides, flavonoids, alkaloids and phenols [24].

This study presents the first report on the antibacterial activities of various plant extract combinations of four Kenyan medicinal plants.

## Methods

### Collection of plant material

*C. sinensis* (leaves) was collected from Shinyalu (Kakamega County). The leaves of *S. didymobotrya* were collected from Kangundo road (Machakos County). The stem bark of *T. asiatica* was collected from Tala (Machakos County) while the leaves of *A. secundiflora* were collected from Matuu (Machakos County). The plants were authenticated at the Department of Botany, University of Nairobi and voucher specimen deposited at the University of Nairobi herbarium. Voucher numbers were allocated as follows: *C. sinensis* (EAO UON 2021/001), *A. secundiflora* (EAO UON 2021/002), *S. didymobotrya* (EAO UON 2021/003) and *T. asiatica* (EAO UON 2021/004). The plant materials were individually air-dried under shade and ground into powder using a laboratory mill [14].

### Extraction procedures

Extraction was separately done using four solvents of different polarities (petroleum ether (PET), dichloromethane (DCM), methanol (MeOH) and water (H_2_O).

### Hot aqueous extraction

About 200 gm of each dried plant powder was separately extracted using 800 ml of distilled water by heating at 60 °C for 30 min. After cooling, the mixture was then filtered through Whatman No.1 filter paper. Reduction was done using a rotary evaporator. The dry aqueous extracts were obtained via lyophilization [26].

### Organic solvent extraction

Approximately 100 gm of each dried powder were separately extracted using 500 ml of petroleum ether, dichloromethane and methanol. Maceration with stirring was done for 72 h at room temperature. The mixture was filtered through Whatman No.1 filter paper. The extracts were then concentrated at 40 °C (for petroleum ether and dichloromethane extracts) and at 65 °C (for methanol extracts) using a rotary evaporator (Heidolph WB2000, Germany) [27]. The organic solvent extracts were further evaporated to dryness at 40 °C in an oven before storing at 4 °C for future use [28].

### Collection of bacterial cultures

Pure bacteria cultures of; *Staphylococcus aureus* ATCC 25,923, *Escherichia coli* ATCC 25,922, *Klebsiella pneumoniae* ATCC 70,063 and *Pseudomonas aeruginosa* 15,422 (from Department of Pharmacy, University of Nairobi) and *MRSA* ATCC 1385 (from Department of Biology, University of Nairobi) were maintained on nutrient broth slants at 4 °C [29]. The standard inoculum suspensions were adjusted to turbidity equivalent to 0.5 McFarland standards and modified to give a density of 1 × 10^6^ cells or spores/ml [29, 30].

### Sterilization and equipment

All the glassware, nutrient media and distilled water used in the antibacterial activity studies were sterilized in an autoclave Memmert Universal oven (Memmet GmbH and Co, KG, Schwabach, Germany) at 121 °C for 15 min before use [28]. The bacteriological wire loop and cork borer were sterilized by flaming using a Bunsen burner flame. All bench work involving use of microorganisms was carried out in a Bioflow laminar flow cabinet (Vermeulen, L.J. BVBA, Westmalle, Belgium) while a Freezer-1 incubator (Analis, Suarlee, Belgium) was used for incubation of the microorganisms [28, 31].

### Preparation of stock solutions

The extracts stock solutions were prepared by dissolving 500 mg in 5 ml of 10% dimethyl sulfoxide (DMSO). Stock solutions of the extract- extract combinations were prepared by combining the two extracts (ratio 1:1) [30]. Gentamicin (30 mg/ 100 ml sterile distilled water) was used as positive control for *Escherichia coli*, *Pseudomonas aeruginosa*, *Klebsiella pneumoniae* [31]. Mupirocin (10 mg/ 100 ml sterile distilled water) was used as positive control for *Staphylococcus aureus* and *Methicillin Resistant Staphylococcus aureus.* A 10% DMSO solution served as the negative control [32, 33].

### Antimicrobial susceptibility testing

Antimicrobial susceptibility testing was done by agar well diffusion method according to the Clinical and Laboratory Standards Institute [34]. The bacterial test organisms were cultured on tryptone soya agar. The nutrient agar was inoculated uniformly with the standardized test organisms. Reservoir wells were formed by cutting out cylindrical plugs from the solidified nutrient agar at equidistant points (30 mm), using a sterile cork borer, to produce wells (diameter 10 mm) [31, 33, 35]. On each petri-dish, the respective wells were separately filled with 100 µl of the stock solutions (10,000 µg/well) single plant extracts, 100 µl plant extract combination, gentamicin 0.3 mg/ml (30 µg/well), mupirocin 0.1 mg/ml (10 µg/well) or 10% DMSO [32]. The inoculated petri-dishes with test solutions in wells were allowed to diffuse for 30 min before overnight (18 h) incubation at 37 °C. All determinations were done in triplicate. The antimicrobial activity was recorded as the diameter (mm) of the of inhibition after incubation [31, 36].

### Minimum inhibitory concentration (MIC) determination

The MICs of the extracts against the test microorganisms were determined by the agar well diffusion method [37–40]. MIC for single extracts and extract combinations that failed to meet minimum activity threshold in the susceptibility studies were not determined [36]. For the single extracts, double serial dilution of the stock solution was carried out resulting in concentration range of 10,000 µg/ml to 78.1 µg/ml [41].

Separate petri-dishes were used for each of the test solutions (single extracts, extract combinations and antibiotic standards). On each petri-dish, the respective wells were separately filled with 100 µl of the respective dilutions [32, 36], The inoculated petri-dishes with test solutions in wells were allowed to diffuse for 30 min before overnight (18 h) incubation at 37 °C. All determinations were done in triplicate. The MIC was determined as the lowest concentration that inhibited visible bacterial growth on the agar subculture [30].

### Determination of fractional inhibitory concentration index

The antibacterial effects of combining selected plant extracts were assessed using the checkerboard method [30]. The fractional inhibitory concentration (FIC) was derived from the lowest concentrations of the extract and the extract in combination permitting no visible growth of the test organisms after incubation [42]. FIC indices were calculated using the formula described [43]: FIC index = (MIC of extract 1 in combination/MIC of extract 1 alone) + (MIC of extract 2 in combination /MIC of extract 2 alone). The following criterion was used in the interpretation of the FIC Index in relation to the mode of plant extract interactions: FICI ≤ 0.5 = synergistic effect; FICI > 0.5 but ≤ 1 = additive effect; FICI > 1, but ≤ 4 = indifferent effect and FICI > 4 = antagonistic effect [43]. The data were analyzed by using MS Excel 2016 and presented as mean ± SD of three replicates. The significance was evaluated by analysis of variance (ANOVA) test and by Tukey’s post hoc multiple comparison test using Statistical Package for the Social Sciences (SPSS) 21.0. Significant differences in the data were established at the 5% level of significance [44].

## Results

Both the single and the combined plant extracts in this study displayed activity against the test bacteria. The patterns of antibacterial activity varied with the plant, test microorganism and the solvent used for extraction. Generally, *C. sinensis* displayed activity against the widest range of microorganisms and the polar extracts from all the four plants demonstrated higher antibacterial activity than the non-polar extracts. For example, the methanol extract of *S. didymobotrya* and that of *C. sinensis* (Table 1) individually displayed low activity against *E. coli* but exhibited an increase in the zone of inhibition in combination (Table 2). This scenario is replicated with the combination of the methanol extract of *A. secundiflora* and methanol *C. sinensis* (Table 2). The combination of dichloromethane extracts of *S. didyobotrya* and *T. asiatica* were not effective in inhibiting *MRSA*. A few (5.26%) of the extract combinations resulted in lower zones of inhibition than the single plant extracts (Tables 1 and 2).

**Table 1:** Table showing mean diameter of zones of inhibition and Minimum inhibitory concentration (MIC) of single plant extracts against *P. aeruginosa, Klebsiella pneumoniae, Escherichia coli, Staphylococcus aureus* and *Methicillin Resistant Staphylococcus aureus*

	*Pseudomonas aeruginosa*		*Klebsiella pneumoniae*		*Escherichia coli*		*Staphylococcus aureus*		*Methicillin Resistant Staphylococcus aureus*	
EXTRACT (100 mg/ ml)	DZI (mm) ± SD	MIC (µg/ well)	DZI (mm) ± SD	MIC (µg/ well)	DZI (mm) ± SD	MIC (µg/ well)	DZI (mm) ± SD	MIC (µg/ well)	DZI (mm) ± SD	MIC (µg/ well)
Aloe MeOH	17.88 ± 0.22	5000	14.60 ± 0.18	5000	13.41 ± 0.18	10,000	14.39 ± 0.21	10,000	11.43 ± 0.12	10,000
Toddalia MeOH	16.11 ± 0.14	5000	13.79 ± 0.02	5000	14.96 ± 0.36	10,000	13.82 ± 0.16	5000	14.07 ± 0.05	2500
Senna MeOH	17.91 ± 0.02	312.5	14.40 ± 0.05	10,000	11.25 ± 0.13	10,000	15.75 ± 0.40	5000	12.03 ± 0.18	10,000
Camelia MeOH	21.67 ± 0.08	312.5	14.01 ± 0.21	5000	13.03 ± 0.04	5000	16.74 ± 0.31	1250	16.57 ± 0.26	1250
Aloe DCM	10.24 ± 0.01	ND	10.15 ± 0.03	ND	10.08 ± 0.02	ND	10.83 ± 0.14	ND	10.13 ± 0.04	ND
Toddalia DCM	16.87 ± 0.23	1250	10.23 ± 0.07	ND	12.55 ± 0.11	ND	11.46 ± 0.15	ND	13.48 ± 0.12	5000
Senna DCM	10.17 ± 0.06	ND	10.38 ± 0.12	ND	10.12 ± 0.03	ND	12.29 ± 0.18	ND	10.04 ± 0.01	ND
Camelia DCM	11.15 ± 0.08	ND	10.00 ± 0.01	ND	10.36 ± 0.04	ND	10.03 ± 0.01	ND	10.08 ± 0.01	ND
Aloe PET	10.30 ± 0.04	ND	10.21 ± 0.04	ND	10.10 ± 0.02	ND	10.65 ± 0.13	ND	10.06 ± 0.02	ND
Toddalia PET	10.22 ± 0.02	ND	10.28 ± 0.02	ND	10.16 ± 0.05	ND	11.61 ± 0.04	ND	11.13 ± 0.03	ND
Senna PET	10.12 ± 0.06	ND	10.32 ± 0.09	ND	10.17 ± 0.09	ND	12.55 ± 0.12	ND	10.10 ± 0.02	ND
Camelia PET	11.04 ± 0.01	ND	10.24 ± 0.03	ND	11.05 ± 0.14	ND	10.11 ± 0.01	ND	10.04 ± 0.01	ND
Aloe H_2_O	11.33 ± 0.25	ND	10.49 ± 0.11	ND	10.04 ± 0.01	ND	10.35 ± 0.19	ND	10.24 ± 0.02	ND
Toddalia H_2_O	10.31 ± 0.16	ND	10.33 ± 0.08	ND	10.10 ± 0.02	ND	11.06 ± 0.02	ND	10.26 ± 0.01	ND
Senna H_2_O	19.72 ± 0.29	312.5	11.39 ± 0.13	10,000	10.42 ± 0.16	10,000	13.12 ± 0.04	10,000	10.50 ± 0.06	10,000
Camelia H_2_O	15.66 ± 0.23	2500	13.48 ± 0.03	2500	17.04 ± 0.24	2500	14.75 ± 0.27	2500	15.05 ± 0.13	2500
DMSO	0.00		0.00		0.00		0.00		0.00	
Gentamycin sulphate (0.3 mg/ ml)	23.24 ± 0.20	4.375	14.00 ± 0.02	8.75	16.02 ± 0.08	8.75				
Mupirocin (0.1 mg/ml)							25.92 ± 0.23	2.315	25.9 ± 0.10	8.75

**Table 2:** Table showing mean diameter of zones of inhibition, Minimum inhibitory concentration (MIC) and Fractional Inhibitory Concentration Index (FICI) of plant extract combinations against *Pseudomonas aeruginosa, Klebsiella pneumoniae, Escherichia coli, Staphylococcus aureus* and *Methicillin Resistant Staphylococcus aureus*

	*Pseudomonas aeruginosa*			*Klebsiella pneumoniae*			*Escherichia coli*			*Staphylococcus aureus*			*Methicillin Resistant Staphylococcus aureus*		
EXTRACT (100 mg/ ml)	DZI* (mm) ± SD	MIC (µg/well)	Σ FICI	DZI* (mm) ± SD	MIC (µg/well)	Σ FICI	DZI* (mm) ± SD	MIC (µg/well)	Σ FICI	DZI* (mm) ± SD	MIC (µg/well)	Σ FICI	DZI* (mm) ± SD	MIC (µg/well)	Σ FICI
Camelia MeOH/Senna MeOH	27.22 ± 0.41	156.25	**1.0** ^**^	14.93 ± 0.35	1250	**0.375** ^*^	14.02 ± 0.03	625	**0.188** ^*^	16.43 ± 0.10	1250	1.25^***^	19.91 ± 0.31	1250	**0.5625** ^*^
Camelia MeOH/Aloe MeOH	22.07 ± 0.38	312.5	1.1^***^	14.19	2500	**1.0** ^**^	14.17 ± 0.22	2500	**0.75** ^**^	14.72 ± 0.34	2500	2.25^***^	16.24 ± 0.22	1250	1.125^***^
Camelia MeOH/Toddalia MeOH	18.35 ± 0.22	625	2.1^***^	13.72 ± 0.16	2500	**1.0** ^**^	10.48 ± 0.17	ND	ND	14.43 ± 0.12	2500	2.5^***^	17.06 ± 0.41	1250	1.5^***^
Camelia MeOH/Toddalia DCM	19.03 ± 0.46	625	2.5^***^	13.51 ± 0.10	ND	ND	10.05 ± 0.01	ND	ND	13.49 ± 0.23	ND	ND	16.28 ± 0.32	1250	1.25^***^
Senna DCM/Toddalia DCM	ND	ND	ND	12.77 ± 0.01	ND	ND	ND	ND	ND	14.33 ± 0.16	ND	ND	10.70 ± 0.06	ND	ND
Camelia H2O/Senna H2O	22.22 ± 0.15	156.25	**0.5625** ^*^	10.36 ± 0.10	ND	ND	10.15 ± 0.04	ND	ND	14.1 ± 0.02	10,000	5^****^	13.96 ± 0.24	1250	**0.625** ^**^
DMSO	0.00			0.00			0.00			0.00			0.00		
Gentamycin sulphate (0.3 mg/ml)	23.24 ± 0.20	4.375		14.00 ± 0.02	8.75		16.02 ± 0.08	8.75							
Mupirocin (0.1 mg/ml)										25.92 ± 0.23	2.315		25.9 ± 0.10	2.315	ND

^*^Synergy ^**^ Additive ^***^Indifferent ^****^Antagonism

The absolute values of the diameter of zones of inhibition (DZI) varied from 10.04 to 27.22 mm (Tables 1 and 2). The minimum inhibitory concentration range for the extract combinations was 10,000 µg/well – 156.25 µg/well and 10,000 µg/well – 1250 µg/well for single extracts (Tables 1 and 2). The ANOVA test indicated significant difference (P < 0.05) in bioactivity between these combinations. The fractional inhibitory concentration indices (FICI) showed that the interactions were synergistic (10.5%), additive (31.6%), indifferent (52.6%), and antagonistic (5.3%). The fractional inhibitory concentration indices (FICI) ranged from 0.5 to 2.5 for *P. aeruginosa*, 0.375 to 1.0 for *K. pneumoniae*, 0.188 to 0.75 for *E. coli*, 1.25 to 5 for *S. aureus* and 0.5625 to 0.625 for *MRSA* strains. The best synergistic interaction (FICI 0.188) appeared with *Camelia* methanol and *Senna* methanol combination against *E. coli* strain.

## Discussion

The polar single extracts had higher activity than the non-polar single extracts. This is in agreement with the findings of a previous studies [36, 45–49]. The methanol crude extracts showed more inhibition than the aqueous extracts (Table 1). This observation is similarly reported in previous studies [50–52]. It is possible that the aqueous crude extracts may contain a lower concentration of antibacterial constituents and this may explain why large quantities of decoctions are taken over a relatively long period to achieve therapeutic success [53, 54].

It is evident that combining some plant extracts improved bioactivities over individual plant extracts. In this study, the polar compounds interacted more synergistically than the non-polar compounds. These findings are comparable to previous studies [7, 10, 45, 55, 56]. Combination drug therapies target multiple pathologic processes thus are capable of suppressing bacterial resistance mechanisms to remedy bacteria [8, 57].

The observed synergistic activity may be explained by the ability of compounds within the plants extracts to interact with one another to improve their solubility, enhance their bioavailability and subsequent antibacterial activity. Possible differences in modes of action of different compounds present in the combined extracts may also result in synergism [58–60]. Pharmacodynamic synergy may have also occurred resulting in different agents regulating either the same or different target in various pathways [61]. The combinations that displayed these positive interactions can be considered as a potential strategy to combat bacterial resistance.

As previously reported elsewhere, non-polar extracts seem less potent than the polar extracts (Tables 1 and 2) [62, 55]. For combinations with non- polar constituents, higher doses but within safety levels can be explored in future [36, 55].

The lower activity in some combinations may be attributed to the respective compounds either neutralizing each other’s activity or forming inactive complexes when in combination [63, 64]. Combination of compounds with minor structural differences that may compete for the same molecular target could also result in antagonism [65]. For the combinations that displayed antagonistic activity, different combination ratios could be further explored [66, 67].

The observed variation in the antibacterial activities for specific plant extract combinations could be due to the differences in chemical composition and concentrations [44, 68]. Some constituents from the plants have reported antibacterial activity through various mechanisms. Ulopterol, a coumarin compound from *T. asiatica* has been shown to inhibit the growth of *K. pneumoniae* and *E. coli* [69]. An alkaloid (chelerythrine) isolated from *T. asiatica* exerts its antibacterial activity via destroying the cell wall and membrane [70].

Tannins present in *C. sinensis* are shown to react with proteins of the bacterial cell wall to form stable water-insoluble components [71]. Flavonoids bind with intracellular proteins as well as soluble proteins present in the bacterial cell walls. Steroids are shown to form complexes with membrane lipids thus resulting in leakage [21, 72, 73]. These compounds present in In *C. sinensis*, may have contributed to the observed antibacterial effect. The exhibited antibacterial activity of *S. didymobotrya* may be due to the presence of alkaloids that are known to interchelate with DNA of both Gram positive and negative bacteria and interfere with cell division [74].

Essential oils have been shown to disrupt the cell wall and lipid bilayer of gram-positive bacteria, resulting in the disarray of metabolic processes and cell lysis [75]. This may account for the antibacterial activity observed in non-polar extracts of *T. asiatica* and *S. didymobotrya* against *S. aureus* and *MRSA.*

In this study, the combination of extracts with a similar phytochemical profile displayed increased bioactivity as in the case of *S. didymobotrya* and *C. sinensis*. This may be due to increased concentrations of the similar antibacterial compounds thus resulting in higher potency.

## Conclusion and recommendations

Plants remain a valuable resource while bioprospecting for novel antimicrobial drugs. The findings of this study support the use of multiple herbs to manage bacterial infections as an appreciable proportion of tested combinations exhibited synergistic and additive properties. To optimize the use of such combinations, there may be need to first standardize the herbal preparations in a bid to ensure efficacy and for safe delivery. Further studies on combination of isolated compounds responsible for the observed antibacterial activity may guide into realization of novel antibacterial agents.

## Data Availability

All relevant data are within the paper and its Supporting Information files. The datasets used and/or analyzed during the current study are available from the corresponding author on reasonable request.

## Abbreviations

ANOVA: Analysis of variance
CLSI: Clinical and Laboratory Standards Institute
DCM: Dichloromethane
DZI: Diameter of zones of inhibition
FICI: Fractional Inhibitory Concentration Index
H_2_O: Water
MeOH: Methanol
MIC: Minimum Inhibitory Concentration
PET: Petroleum ether
